# Obesity and abdominal hernia in ambulatory patients, 2018–2023

**DOI:** 10.1007/s10029-024-03034-8

**Published:** 2024-05-25

**Authors:** H. Zelicha, D. S. Bell, D. Chen, Y. Chen, E. H. Livingston

**Affiliations:** 1 Department of Surgery, Faculty of Health Sciences, UCLA School of Medicine, CHS 74-121, Los Angeles, CA 90095 USA; 2 Department of Medicine, Division of General Internal Medicine, UCLA, Los Angeles, CA USA; 3 Informatics Program of the UCLA Clinical and Translational Science Institute (CTSI), UCLA, Los Angeles, CA USA

**Keywords:** Abdominal, BMI, Hernia, Obesity

## Abstract

**Purpose:**

To determine the relationship between abdominal hernia and obesity. Although obesity is frequently cited as a risk factor for abdominal hernia, few studies have confirmed this association (Menzo et al. Surg Obes Relat Dis 14:1221–1232. 10.1016/j.soard.2018.07.005, 2018).

**Methods:**

A cross-sectional study of primary care ambulatory patients aged older than 16 years treated at UCLA Health from 01/01/2018 to 06/06/2023. Abdominal hernia was identified by clinic encounter ICD-10 codes (K40–K46).

**Results:**

There were 41,703 hernias identified among 1,362,440 patients (306.1 per10,000) with a mean age of 62.5 ± 16.1 years, and 57.6% were men. Nearly half (44.7%) of all abdominal hernias were diaphragmatic. There was an approximately equal distribution of the ventral (28.7%) and inguinal (24.3%) hernia. Each hernia type had a different relationship with obesity: The odds of having a ventral hernia increased with BMI in both sexes: BMI 25–29.9 kg/m^2^ odds ratio (OR) = 1.65, (CI 1.56–1.74); BMI 30–39.9 kg/m^2^ OR = 2.42 (CI 2.29–2.56), BMI 40–49.9 kg/m^2^ OR = 2.28 (CI 2.05–2.54) and BMI >  = 50 kg/m^2^ OR = 2.54 (CI 2.03–3.17) all relative to normal BMI. In contrast, the odds of having an inguinal hernia decreased with obesity relative to normal weight [obesity (BMI 30–39.9 kg/m^2^): OR = 0.60 (CI 0.56–0.65)], morbid obesity (BMI 40–49.9 kg/m^2^): OR = 0.29 (CI 0.23–0.37). The OR for diaphragmatic hernia peaks with obesity in women and overweight status in men but was found to decrease with morbid obesity [OR = 1.18 (CI 1.07–1.30)]. There was no significant difference between men and women in the prevalence of femoral hernia (men: 0.7/per10,000, women: 0.9/per10,000, *p* = 0.19).

**Conclusions:**

The relationship between hernia and obesity is complex with some hernias decreasing in prevalence as obesity increases. Further research is needed to better understand this paradoxical relationship.

**Supplementary Information:**

The online version contains supplementary material available at 10.1007/s10029-024-03034-8.

## Introduction

Obesity is commonly cited as a risk factor for abdominal hernia [1]. Despite this widely cited assertion, when stated in the literature the claim is rarely accompanied by referenced articles with actual investigational findings. Although there is a very large literature describing the influence of obesity on hernia recurrence, there is a paucity of original data examining the relationship between obesity and primary abdominal hernia [2].

Hernias are thought to be caused by metabolic factors influencing fascia integrity and mechanical pressure from within the abdomen [2, 3]. Intrabdominal pressure should increase with obesity [3], and that pressure should generally be higher in men than women because of their propensity for central obesity as opposed to women who tend to have more peripheral fat distributions [4]. Taken together, these factors would predict an increase in ventral hernia with increasing obesity, an association that should be more pronounced in men as compared with women.

We hypothesized that there will be an increase in diaphragmatic, ventral, and inguinal hernia with increasing levels of obesity. Furthermore, because men tend to have more central obesity, we hypothesized that men will have more hernias and more of an effect of obesity on abdominal hernia.

## Methods

### Patient population

All ambulatory patients diagnosed with a hernia, aged 16 and above seen at UCLA Heath primary care clinics from 01/01/2018 to 06/06/2023 were included. The presence of abdominal hernia was determined from ICD-10 diagnostic codes (K40–K46) assigned to ambulatory clinic encounters. We only examined records that had a hernia diagnostic code as the primary diagnosis (Supplemental Data 1). The final analytic samples included 41,703 patients with hernia out of 1,361,440 total UCLA patients who had at least one ambulatory encounter in the study period. The study was approved by the UCLA IRB (IRB# 23–000174).

### Clinical data

Age, sex, race, and ethnicity were extracted from the UCLA Epic Clarity EHR database that includes all encounters at UCLA after the removal of restricted patients such as celebrities. We divided the hernia diagnoses into the following groups based on ICD-10 encounter diagnoses: Diaphragmatic (K44.9), ventral (umbilical, incisional, ventral, other, unspecified) (K42, K43, K45, K46), and inguinal (including femoral) (K40, K41) (Supplemental Data 1). We further stratified the groups into those with or without obstruction or gangrene based on ICD-10 codes (Supplemental Data 1 shows all individual codes used). In the case of multiple hernia diagnoses, the first diagnosis was presented.

Every measure of BMI available in the last 5 years was also obtained. The BMI assigned to each individual patient was measured within 6 months of the hernia diagnosis. A mean BMI value was calculated in case of multiple BMI observations in the same time frame of 6 months. BMI values less than 13 and above 100 were assumed spurious and excluded from the analysis. BMI was categorized into the following groups: underweight: BMI ≤ 18.5 kg/m^2^, normal weight: 18.5–24.9 kg/m^2^, overweight: 25–29.9 kg/m^2^, obesity: 30–39.9 kg/m^2^, morbid obesity: 40–49.9 kg/m^2^, and super obesity: BMI ≥ 50.

### Statistical analysis

**Fig. 1:**
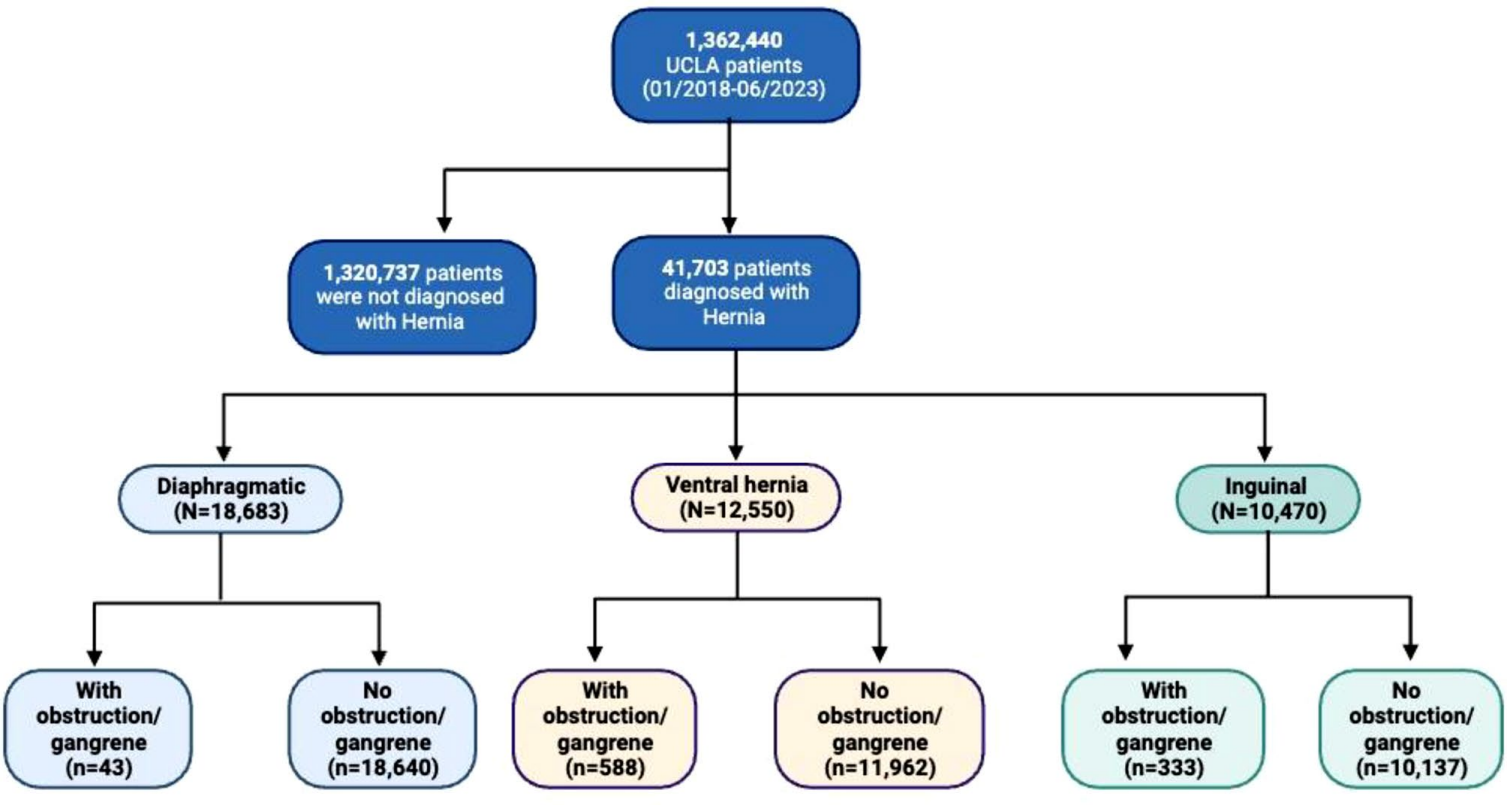
Flowchart of study patients

The primary aim was to examine the prevalence of abdominal hernia and its relationship to obesity. A flow chart of the data processing study is presented in Fig. 1. Continuous variables are presented as the means (standard deviations) or medians and IQR. Nominal variables are expressed as numbers and percentages. The Kolmogorov–Smirnov test was used to determine whether variables were normally distributed. The differences across the groups were tested using ANOVA, *T* test, the Kruskal–Wallis test, the Mann–Whitney test, or the Chi-square statistic for nominal variables. In addition, the differences between groups are presented as standardized mean differences (SMD). By convention, small differences (e.g., small effect sizes) have SMD ≤ 0.2, medium 0.5, and the differences or effects considered large when the SMD > 0.8 [5]. The Kendall tau correlation was used to examine the trend of *P*. Multiple comparisons were adjusted using the Bonferroni correction. We used logistic regression models to assess the odds ratio of hernia across BMI categories in men and women populations, adjusted for various factors that might contribute to hernia formation. In addition, to test the hypothesis that inguinal hernias are the most common groin hernia in both men and women but that femoral hernias are more common in women than men, we compared the sex-stratified prevalence of these hernias. Statistical significance was set at a two-sided α = 0.05; all statistical analyses were performed with the R studio program version 4.3.1.

## Results

### Population characteristics

**Fig. 2:**
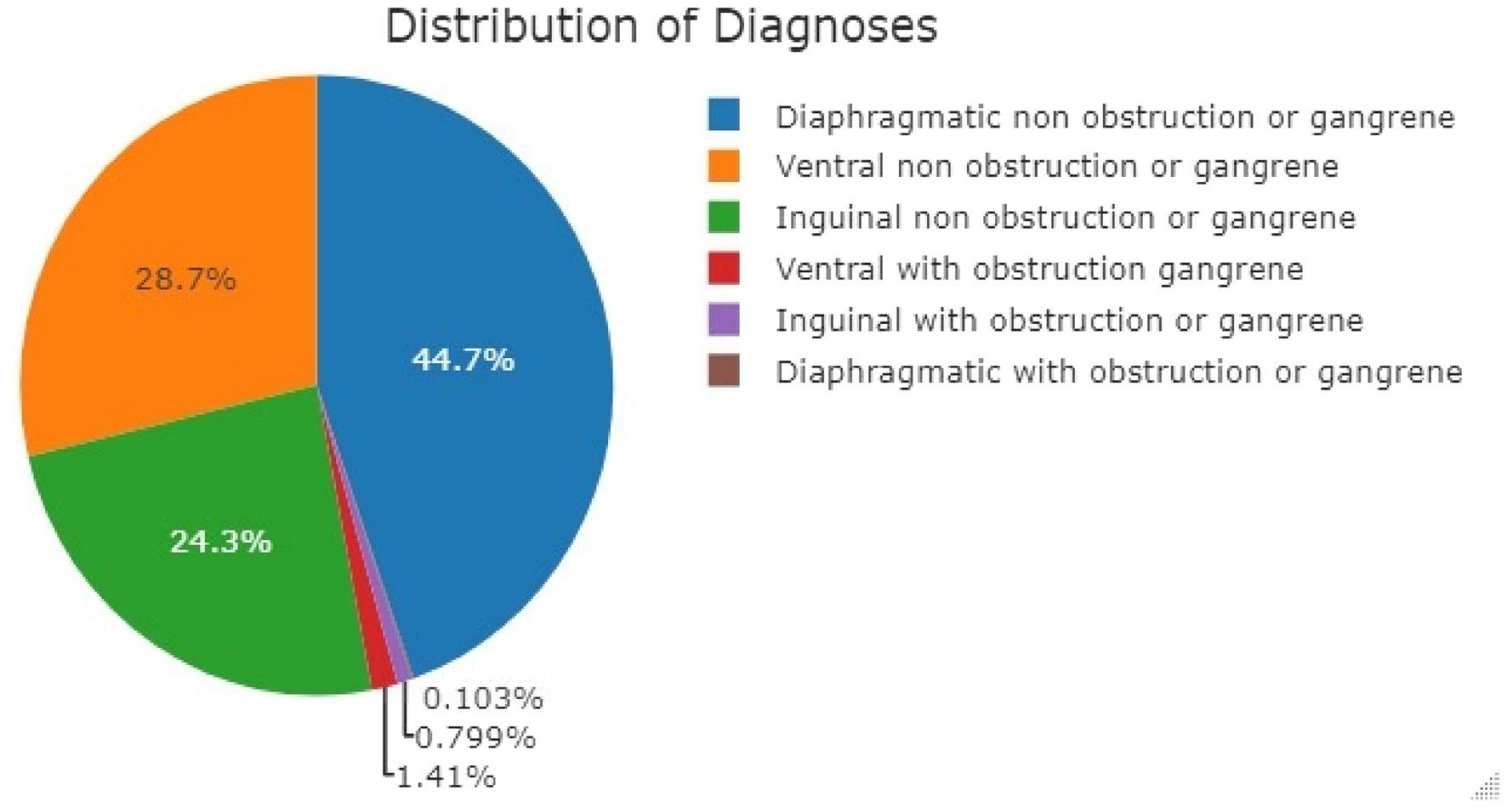
Distribution of hernias by type

There were 41,703 hernias identified among 1,362,440 patients (306.1 per 10,000) who had a mean age of 62.5 ± 16.1 years, 57.6% men, mean BMI = 27.6 ± 5.8 kg/m^2^, and 14.7% were in Hispanic patients (Table 1, Supplemental Fig. 1). Diaphragmatic hernias accounted for nearly half (44.7%) of all hernias identified in routine clinic encounters (Fig. 2). There was an approximately equal distribution of the ventral and inguinal hernia (28.7% with ventral hernia, 24.3% with inguinal hernias) (Fig. 2). Hernias complicated by obstruction or gangrene was uncommon (< 2.5%) but, when present, was associated with increased age (inguinal: with obstruction/gangrene 66.8 ± 16.4 years, no obstruction/gangrene 61.3 ± 16.4 years; ventral: with obstruction/gangrene 61.9 ± 15.8 years, no obstruction/gangrene 59.2 ± 15.4 years) (Supplemental Table 1; Supplemental Fig. 2). Only 2.1% of the hernias were recurrent, with a higher rate among men (3.2%) than women (0.6%; *p* < 0.001, SMR = 0.09) (Table 1).

**Table 1:** Characteristics of the study population across sex*:

	Entire (*N* = 41,703)	Men *N* = (24,005)	Women *N* = (17,693)	p value between groups**	Standardized mean difference (SMD)
Age, year	62.5 ± 16.1	61.5 ± 15.9	63.7 ± 16.4^**^	< 0.001	− 0.14
Hispanics, *n* (%)	6146 (14.7)	3152 (13.1)	2993 (16.9)^**^	< 0.001	0.07
Race				< 0.001	0.06
White, *n* (%)	19,730 (47.4)	11,486 (47.8)	8243 (46.6)^a^	< 0.001	0.23
Black, *n* (%)	2321 (5.6)	1093 (4.6)	1226 (6.9)^a^	0.006	0.03
Asian, *n* (%)	2113 (5.1)	1134 (4.7)	979 (5.5) ^a^	< 0.001	0.03
American Indian or Alaska native, *n* (%)	191 (0.5)	115 (0.5)	76 (0.4)^a^	0.005	0.03
Unknown/refused to answer, *n* (%)	14,889 (35.7)	8782 (36.6)	6106 (34.5)^a^	< 0.001	0.22
Middle Eastern or north African, *n* (%)	883 (2.1)	543 (2.3)	340 (1.9)^a^	< 0.001	0.07
Multiple races, *n* (%)	1451 (3.5)	780 (3.2)	670 (3.8)^a^	0.004	0.03
Native Hawaiian or other pacific islander, *n* (%)	74 (0.2)	36 (0.1)	38 (0.2)	0.8	0
Body mass index, kg/m^2^	27.6 ± 5.8	27.5 ± 5.0	27.7 ± 6.8	0.02	− 0.03
Body mass index categories				< 0.001	0.18
Underweight, *n* (%)	594 (1.9)	172 (1.0)	422 (3.2)^a^	< 0.001	0.10
Normal weight, *n* (%)	10,415 (33.5)	5567 (31.0)	4847 (37.0)^a^	< 0.001	0.07
Overweight, *n* (%)	11,555 (37.2)	7761 (43.2)	3794 (29.0)^a^	< 0.001	0.37
Obesity, *n* (%)	7371 (23.7)	4077 (22.7)	3293 (25.2)^a^	< 0.001	0.09
Morbid obesity, *n* (%)	971 (3.1)	348 (1.9)	622 (4.8)^a^	< 0.001	0.09
Super morbid, *n* (%) obesity	151 (0.5)	45 (0.3)	106 (0.8)^a^	< 0.001	0.05
Weight (kg)	80.5 ± 19.5	86.4 ± 17.6	72.3 ±19.0^**^	< 0.001	0.77
Height (m2)	1.71 ± 0.1	1.77 ± 0.1	1.62 ± 0.1^**^	< 0.001	2.00
Recurrence, *n* (%)	867 (2.1)	764 (3.2)	103 (0.6)^**^	< 0.001	0.09

*Values are presented as mean ± standard deviation or median (IQR) for continuous variables and as number and % for categorical variables

**p value according to t-test/Mann-Whitney test for continuous variables and Chi-square for categorical variables

^a^Bonferroni correction was used. BMI was categorized into the following groups: underweight: BMI ≤ 18.5 kg/m^2^, normal weight: 18.5–24.9 kg/m^2^, overweight: 25–29.9 kg/m^2^, obesity: 30–39.9 kg/m^2^, morbid obesity: 40–49.9 kg/m^2^, super obesity: BMI ≥ 50 kg/m^2^

### Hernia and BMI

**Fig. 3:**
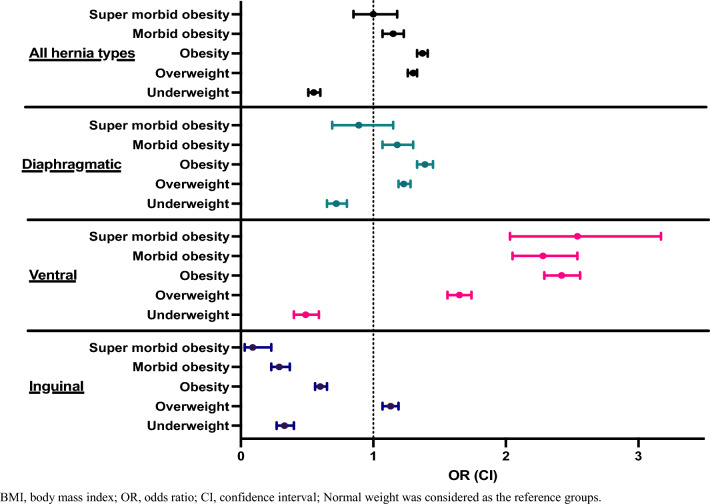
Forest plot of abdominal hernias across BMI categories. Odds ratios are shown relative to normal weight BMI categories. *BMI* body mass index, *OR* odds ratio, *CI* confidence interval

The mean BMI of patients who had inguinal hernia was significantly lower as compared with ventral and diaphragmatic hernia. Of the various hernias, the mean BMI for ventral hernia was the highest (inguinal: 26.1 ± 4.3 kg/m^2^, ventral: 29.0 ± 6.3 kg/m^2^; diaphragmatic: 27.6 ± 5.9 kg/m^2^, *p* < 0.001) (Supplemental Table 2). The odds of having a ventral hernia consistently increased with BMI as compared with normal weight: overweight: OR = 1.65, CI 1.56–1.74; obesity: OR = 2.42 (CI 2.29–2.56), morbid obesity: OR = 2.28 (CI 2.05–2.54) and super morbid obesity: OR = 2.54 (CI 2.03–3.17) (Fig. 3, Supplemental Tables 2 and 3). However, the odds of having inguinal hernia peaked with the overweight condition and then the odds decreased with obesity and morbid obesity [obesity: OR = 0.60 (CI 0.56–0.65), morbid obesity: OR = 0.29 (CI 0.23–0.37) (Fig. 3, Supplemental Tables 2 and 3)]. The odds ratio for diaphragmatic hernia peaked with obesity but decreased with morbid obesity [OR = 1.18 (CI 1.07–1.30) (Fig. 3, Supplemental Tables 2 and 3)]. There were no significant differences in BMI for hernia with and without complications (diaphragmatic no obstruction/gangrene: 27.6 ± 5.9 kg/m^2^, diaphragmatic with obstruction/gangrene: 27.4 ± 6.0 kg/m^2^, ventral no obstruction/gangrene: 29.0 ± 6.3 kg/m^2^, ventral with obstruction/gangrene: 28.9 ± 7.2 kg/m^2^; inguinal no obstruction/gangrene: 25.9 ± 4.5, inguinal no obstruction/gangrene: 26.1 ± 4.3 kg/m^2^; *p* > 0.05 Bonferroni correction) (Supplemental Table 1).

### Hernia prevalence across sex

**Fig. 4:**
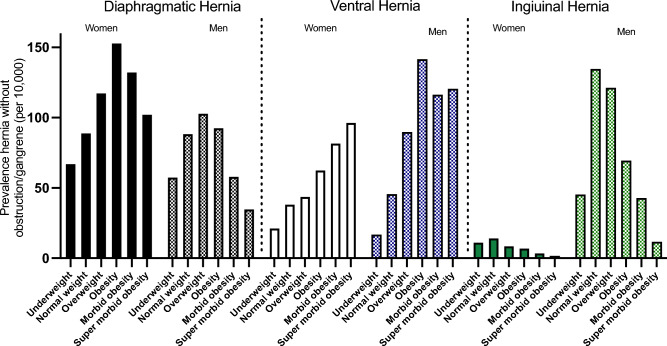
The prevalence of abdominal hernias stratified by BMI categories and sex

The overall prevalence of hernia in men was 394.1 per 10,000 and 227.8 per 10,000 for women. The odds of having a hernia across BMI categories differed between men and women: women had a relatively consistent increase in OR with higher BMI categories, whereas in men, the odds reached their peak in overweight [OR: 1.18 (CI 1.14–1.22)], and then consistently decrease with each BMI category (Fig. 3, Supplemental Tables 4 and 5). Men have a higher prevalence of ventral hernia and inguinal hernia vs. women (ventral: men 85.36/per 10,000, women 45.53/per 10,000; inguinal hernia: men 110.38/per 10,000, women 10.73/per10,000). However, women had a slightly higher prevalence of diaphragmatic hernia than men (men: 93.48/per 10,000; women: 109.65/per 10,000) (Supplemental Tables 4 and 5). The peak of diaphragmatic hernia prevalence was reached in obesity among women and overweight in men, and consistently decreased in morbid and super morbid obesity. The prevalence of ventral hernia consistently increased across BMI categories in both men and women. However, among men, the highest prevalence observed was in the obesity category. The prevalence of inguinal hernia was significantly higher in men and decreased in obesity (Fig. 4, Supplemental Tables 4 and 5). In addition, we compared the prevalence of inguinal hernias and femoral hernias separately and stratified by sex and found that men had a significantly higher prevalence of inguinal hernia as compared with women (men: 146.4/per 10,000 women: 14.2 per/10,000; *p* < 0.001) (Supplemental Fig. 3). However, there was no significant difference between men and women in the prevalence of femoral hernia (men: 0.7/per 10,000, women: 0.9/per 10,000, *p* = 0.19) (Supplemental Fig. 4).

## Discussion

In our study among over 41,000 patients seen in ambulatory clinics for a primary diagnosis of hernia from 2018 to 2023, the overall prevalence of hernia was almost twice that for men as compared with women, mainly driven by the much lower inguinal hernia prevalence in women. Most patients with hernia exceeded normal BMI. There was a long, right tail in the distribution for women, suggesting greater increases in abdominal hernia risk for women as obesity increases relative to men. Each hernia type was found to have a different relationship with obesity and sex. Increased BMI was associated with an increased prevalence of ventral hernia and a decreased prevalence of inguinal hernia. However, diaphragmatic hernia, which was the most common hernia type, decreased in prevalence with morbid obesity. There was no association between BMI and the hernia complications of obstruction and gangrene. We studied an ambulatory population which might explain why we observed so few cases of obstruction or gangrene (< 2.5%), complications that would be expected to be encountered in hospital settings. Given the small numbers we could not reliably separate obstruction from gangrene in our analysis.

Diaphragmatic hernia has its own ICD-10 code that is distinct from gastroesophageal reflux disease (GERD). Using ICD-10 codes entered as the primary diagnosis in ambulatory care encounters, we found that about 50% of all abdominal hernias were coded as diaphragmatic. Although there are numerous types of diaphragmatic hernia, the coding system aggregates numerous types of hernia into a single code. Even though this limits how granular an analysis is possible using clinic encounter codes, the code assessed in this work (ICD10 K44) is what primary care clinicians use and use it very frequently.

The prevalence of diaphragmatic hernia was associated with BMI but nonlinearly. Diaphragmatic hernia was more common in women than men and increased in prevalence with increasing BMI, peaking at obese BMI for women and overweight for men. The prevalence of diaphragmatic hernia fell as BMI increased beyond 40 kg/m^2^. These findings are similar to those of symptomatic GERD in the Nurses’ Health Study where GERD increased with increasing BMI peaking at BMI = 30 kg/m^2^ and then remaining constant with higher BMI [6].

Our results differ from prior reports of the prevalence of diaphragmatic hernia and obesity. A series of preoperative, obese bariatric surgery patients who underwent routine upper GI series examinations was found to have a prevalence of anatomical hiatal hernia of 37% [7]. In the Nurses’ Health Study, 10,545 women were surveyed with 22% reporting GERD symptoms (presumably related to diaphragmatic hernia) with about ½ of these noting that GERD was moderately severe [6]. In contrast, we found a prevalence of diaphragmatic hiatal hernia of 0.5% in the smallest patients to about 1.5% in the largest. The lower prevalence in our series relative to others can be explained by identifying diaphragmatic hernia from clinic encounter codes where the primary reason for the visit was diaphragmatic hernia. Most diaphragmatic hernias are asymptomatic, and only a small proportion of them result in symptoms significant enough to become the primary diagnosis in seeking medical care. The very low prevalence of symptomatic diaphragmatic hernia in our study suggests that diaphragmatic hernias should not be repaired unless associated with symptoms.

The current study confirms that the prevalence of inguinal hernia in men is 10 × that for women but that the prevalence of femoral hernia is slightly greater in women (0.93 per/10,000) than in men (0.72 per/10,000) with no significant difference. Prior reports of these sex-based relationships were mostly from Scandinavian populations of patients undergoing hernia surgery [8–11]. We confirm that these relationships hold true in a US-based, very heterogenous patient population of outpatients irrespective of hernia repair status.

There is limited epidemiological evidence on the relationship between hernia prevalence and obesity. Prior literature suggests that ventral hernias increase with obesity [2]. However, numerous studies have shown an inverse relationship between groin hernia and BMI in men [12–21] and in women [20–22]. Obesity is associated with increased intraabdominal pressure [23]. Increased pressure pushing structures against the abdominal wall can explain why, in general, there is an increase in diaphragmatic and ventral hernias with obesity. It does not explain why inguinal hernia is less frequent with obesity. The inguinal canal in men is a natural opening in the abdominal wall, necessary to allow the spermatic cord structures to pass from inside to outside the abdominal cavity. When the abdominal wall is tensed, groin hernia is prevented by the conjoint tendon stretching and being displaced towards the inguinal ligament, closing the gap that is normally present in the inguinal canal [18]. One hypothesis for why inguinal hernia is less frequent in obese individuals is that fat accumulations in groin tissues close the gap between the conjoint tendon and inguinal ligament [15]. Alternatively, fat accumulations within intraabdominal structures such as the omentum and mesentery prevent them from herniating through the groin [15]. One possible explanation for why inguinal hernia is less frequent in the obese in our cohort of patients seeking medical care for hernia is that obesity interferes with discovery of groin hernia in the obese. This is unlikely given that ventral hernia is more frequent in the obese yet has the same potential for obesity itself obscuring the presence of hernia.

None of the cited studies could explain why this occurs. Moreover, we expected to find an increase in ventral and diaphragmatic hernia that paralleled increasing BMI with a prevalence that was higher in men than women. Men did have an increase in the prevalence of ventral hernia that increased in prevalence from underweight to the obese state but then leveled off at higher levels of obesity. Women displayed a monotonic increase in ventral hernia development with increasing levels of obesity. In contrast to the observation for ventral hernia, women had a higher prevalence of diaphragmatic hernia than men. Both men and women had increasing numbers of diaphragmatic hernias with increasing obesity, but the prevalence decreased in the morbid obesity categories. The prevailing hypothesis is that obesity results in less hernia detection. Further studies involving imaging could be the key to answering this question.

The association between hernia and obesity could be explained by intra-abdominal pressure. Tissue strength is a complex interplay of factors, based on genetic components and the intricate arrangement of collagen [2, 3]. Moreover, the tension in the abdominal wall can be affected by intra-abdominal pressure and central obesity [2, 3]. In general, men have more abdominal obesity than women, which is accompanied by a higher accumulation of visceral adipose tissue, that increases the amount of pressure on the structures encasing the abdominal cavity: the abdominal wall and diaphragm [4]. In addition, visceral adipose tissue previously showed higher lipogenic and lipolytic activities and produced more pro-inflammatory cytokines, while subcutaneous tissue, which is higher proportion in women, is the main source of leptin and adiponectin [24]. This increased inflammation can also potentially exert damage to the abdominal tissue.

Our study has some limitations. (1) Our data were a convenience sample of data available from the UCLA health system and may not reflect other populations. (2) Although our study had a very large sample of patients studied over the course of 5.5 years, it was limited by being retrospective and because it relied on diagnostic coding and not on objective signs or tests for hernia. This resulted in an underestimation of the true prevalence of hernia because clinicians enter codes for clinical encounters only when the disease is clinically important and causes patients to seek medical care. (3) We found a very low recurrence rate that could have underestimated the true prevalence. (4) BMI does not reflect the body composition. Central rather than peripheral obesity will more profoundly affect intra-abdominal pressure and, therefore, abdominal hernia rates. (5) There are numerous factors contributing to abdominal hernia development including congenital abnormalities, trauma and, for incisional hernia, SSI, increasing age and smoking. These were not examined in the current study as its intent was to provide primary data regarding the obesity–abdominal hernia relationship. This study has clinically important results, and it is one of the first studies that analyze the association of obesity with all types of abdominal hernia.

In conclusion, ventral hernias are more common in obese patients, and increases in BMI are related to increased prevalence of ventral and diaphragmatic hernia. Obesity reduced the prevalence of inguinal hernias. BMI was not related to hernia complications.

### Supplementary Information

Below is the link to the electronic supplementary material.

Supplementary file1 (DOCX 295 KB)

## Declarations

### Data availability

All data collected for this research are reported in the manuscript and accompanying supplement. The data used for this research was obtained from patients receiving care at UCLA and because of privacy concerns is not available to the public. 
