# Supplementary material for: Obesity and abdominal hernia in ambulatory patients, 2018–2023
Source: Hernia. 2024 May 25;28(4):1317–24. doi: 10.1007/s10029-024-03034-8 (PMC11297064; doi:10.1007/s10029-024-03034-8)
Supplement: Supplementary file 1 — Supplementary file1 (DOCX 295 KB) [file 10029_2024_3034_MOESM1_ESM.docx]

**Online Supplements**

Supplemental Data 1. Abdominal hernia as coded by ICD-10 in ambulatory clinic encounters divided into the following groups:

1. Diaphragmatic
   1. Uncomplicated – K44.9 Diaphragmatic hernia without obstruction or gangrene
   2. Complicated -
   3. 44.1 Diaphragmatic hernia with gangrene

44.0 Diaphragmatic hernia with obstruction without gangrene

1. Ventral hernia (K42 Umbilical hernia, K43 Ventral hernia, K45 Other abdominal hernia, K46 Unspecified abdominal hernia)
   1. Uncomplicated
      1. K42.9 Umbilical hernia without obstruction or gangrene
      2. K43.9 Ventral hernia without obstruction or gangrene
      3. K45.8 Other specified abdominal hernia without obstruction or gangrene
      4. K46.9 Unspecified abdominal hernia without obstruction or gangrene
      5. K43.2 Incisional hernia without obstruction or gangrene
      6. K43.5 Parastomal hernia without obstruction or gangrene
   2. Complicated
      1. K42.0 Umbilical hernia with obstruction, without gangrene
      2. K42.1 Umbilical hernia with gangrene
      3. K43.6 Other and unspecified ventral hernia with obstruction, without gangrene
      4. K43.7 Other and unspecified ventral hernia with gangrene
      5. K45.0 Other specified abdominal hernia with obstruction, without gangrene
      6. K45.1 Other specified abdominal hernia with gangrene
      7. K46.0 Unspecified abdominal hernia with obstruction, without gangrene
      8. K46.1 Unspecified abdominal hernia with gangrene
      9. K43.0 Incisional hernia with obstruction, without gangrene
      10. K43.1 Incisional hernia with gangrene
      11. K43.3 Parastomal hernia with obstruction, without gangrene
      12. K43.4 Parastomal hernia with gangrene
2. Inguinal hernia (K40 Inguinal hernia)
   1. Uncomplicated
      1. K40.20 Bilateral inguinal hernia, without obstruction or gangrene, not specified as recurrent
      2. K41.20 Bilateral femoral hernia, without obstruction or gangrene, not specified as recurrent
      3. K40.90 Unilateral inguinal hernia, without obstruction or gangrene, not specified as recurrent
      4. K40.21 Bilateral inguinal hernia, without obstruction or gangrene, recurrent
      5. K40.91 Unilateral inguinal hernia, without obstruction or gangrene, recurrent
      6. K41.21 Bilateral femoral hernia, without obstruction or gangrene, recurrent
      7. K41.91 Unilateral femoral hernia, without obstruction or gangrene, recurrent
      8. K41.90 Unilateral femoral hernia, without obstruction or gangrene, not specified as recurrent
   2. Complicated
      1. K40.00 Bilateral inguinal hernia, with obstruction, without gangrene, not specified as recurrent
      2. K40.10 Bilateral inguinal hernia, with gangrene, not specified as recurrent
      3. K40.30 Unilateral inguinal hernia, with obstruction, without gangrene, not specified as recurrent
      4. K40.40 Unilateral inguinal hernia, with gangrene, not specified as recurrent
      5. K41.00 Bilateral femoral hernia, with obstruction, without gangrene, not specified as recurrent
      6. K41.10 Bilateral femoral hernia, with gangrene, not specified as recurrent
      7. K41.30 Unilateral femoral hernia, with obstruction, without gangrene, not specified as recurrent
      8. K41.40 Unilateral femoral hernia, with gangrene, not specified as recurrent
      9. K40.01 Bilateral inguinal hernia, with obstruction, without gangrene, recurrent
      10. K40.11 Bilateral inguinal hernia, with gangrene, recurrent
      11. K40.31 Unilateral inguinal hernia, with obstruction, without gangrene, recurrent
      12. K40.41 Unilateral inguinal hernia, with gangrene, recurrent
      13. K41.31 Unilateral femoral hernia, with obstruction, without gangrene, recurrent
      14. K41.41 Unilateral femoral hernia, with gangrene, recurrent

Supplemental Figure 1. Distribution of hernia by BMI, stratified by sex. The red lines represent the bounds of normal BMI.


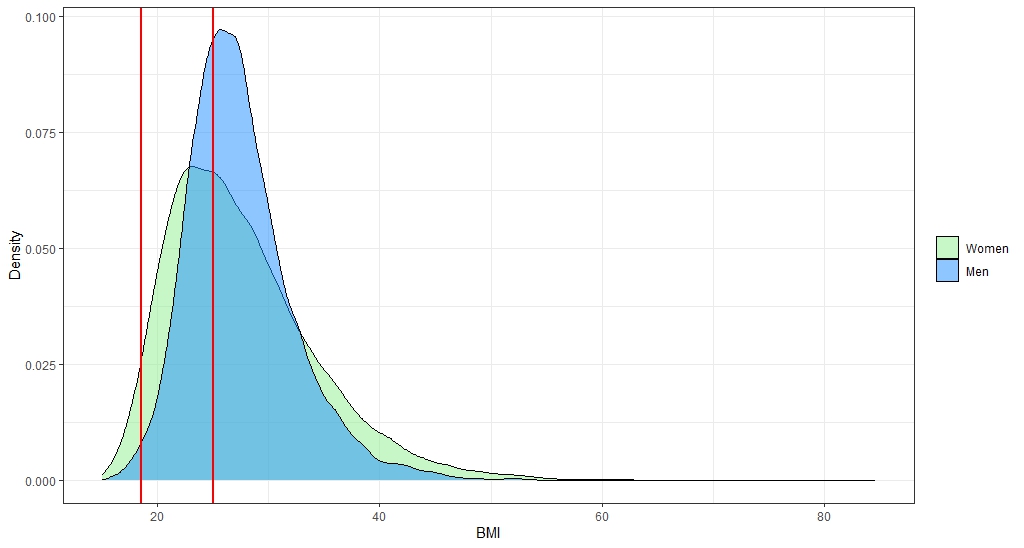


Most patients with hernia fall above the normal range for BMI. There is a long, right tail in the distribution for women, suggesting that there is a greater increase in abdominal hernia risk for women as obesity increases relative to men.

Supplemental Figure 2. BMI and abdominal hernias


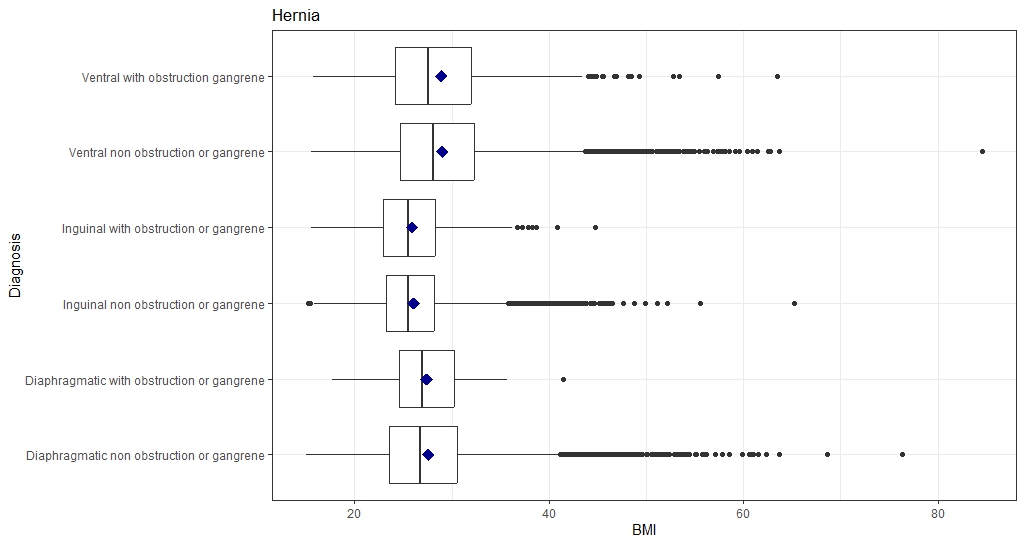


Supplemental Figure 3. The prevalence of inguinal hernia across sex

Supplemental Figure 4. The prevalence of femoral hernia across sex

**Supplemental Table 1**: Characteristics of the study population across different hernia types*:

|  | **Entire**  **(N=41,703)** | **Diaphragmatic**  **(N=18,683)** | | **Ventral Hernia**  **(N=12,550)** | | **Inguinal Hernia**  **(N=10,470)** | | **p value between groups**** | **Standardized Mean Difference (SMD)** |
| --- | --- | --- | --- | --- | --- | --- | --- | --- | --- |
|  |  | **With obstruction/**  **gangrene**  **(n=43)** | **No obstruction/**  **gangrene**  **(n=18,640)** | **With obstruction/**  **gangrene (n=588)** | **No obstruction/**  **gangrene**  **(n=11,962)** | **With obstruction/**  **gangrene**  **(n=333)** | **No obstruction/**  **gangrene**  **(n=10,137)** |  |  |
| **Age, year** | 62.5 ±16.1 | 61.5±21.0 | 65.1±16.0 **^a,c,d^** | 61.9±15.8 | 59.2±15.4**^i^** | 66.8±16.4**^g,h^** | 61.3±16.4 **^e,j^** | **<0.001** | **0.03** |
| **Men, n (%)** | 24,005 (57.6) | 16 (37.2) | 7,612 (40.8) | 304 (51.7) | 6836 (57.2) | 275 (82.6) | 8,962(88.4) | **<0.001** | **0.39** |
| **Hispanics, n (%)** | 6,146 (14.7) | 7(16.3) | 2493(13.4) | 149(25.3) | 2225(18.6) | 47 (14.1) | 1,225(12.1) | **<0.001** | **0.07** |
| **Race** (n=41,652) | | | | | | | | **<0.001** | **0.04** |
| **White, n (%)** | 19,730 (47.4) | 25(58.1) | 8994(48.3) | 260 (44.2) | 5503 (46.1) | 182(54.7) | 4766(47.1) | **<0.001** |  |
| **Black, n (%)** | 2,321  (5.6) | 3(7.0) | 1053(5.7) | 50 (8.5) | 762(6.4) | 26(7.8) | 427(4.2) | **<0.001** |  |
| **Asian, n (%)** | 2,113  (5.1) | 0 (0) | 1177(6.3) | 20 (3.4) | 416(3.5) | 8(2.4) | 492(4.9) | **<0.001** |  |
| **American Indian or Alaska native, n (%)** | 191  (0.5) | 0 (0) | 65(0.3) | 3 (0.5) | 77(0.6) | 1(0.3) | 45(0.4) | **<0.001** |  |
| **Unknown/**  **refused to answer, n (%)** | 14,889(35.7) | 15 (34.9) | 6244(33.5) | 224 (38.1) | 4494(37.6) | 105(31.5) | 3807(37.6) | **<0.001** |  |
| **Middle Eastern or north African, n (%)** | 883  (2.1) | 0 (0) | 414(2.2) | 8 (1.4) | 224(1.9) | 7(2.1) | 230(2.3) | **<0.001** |  |
| **Multiple races, n (%)** | 1,451  (3.5) | 0 (0) | 640(3.4) | 22 (3.7) | 453(3.8) | 4(1.2) | 332(3.3) | **<0.001** |  |
| **Native Hawaiian or other pacific islander, n (%)** | 74(0.2) | 0 (0) | 41 (0.2) | 1 (0.2) | 17(0.1) | 0 (0) | 15(0.1) | **<0.001** |  |
| **Body mass index, kg/m^2^** | 27.6 ±5.8 | 27.4 ±6.0 | 27.6±5.9**^a,b,c,d^** | 28.9±7.2 | 29.0±6.3 | 25.9±4.5 **^g,h^** | 26.1 ±4.3 **^e,f^** | **<0.001** | **0.03** |
| **Weight (kg)** | 80.5±19.5 | 78.2±18.7 | 77.4 ±19.2**^a,c,d^** | 83.2±22.6 | 85.3±21.5 | 78.4±17.2 **^g^** | 80.4±16.1 **^e^** | **<0.001** | **0.03** |
| **Height (m^2^)** | 1.71±0.1 | 1.70±0.1 | 1.67±0.1**^a,b,c,d^** | 1.69±0.1 | 1.71±0.1 **^i^** | 1.74±0.1**^g,h^** | 1.76±0.1**^e,f^** | **<0.001** | **0.09** |
| **Recurrence, n(%)** | 867 (2.1) | 0 | 0 | 0 | 0 | 42(12.6) | 825(8.1)^j^ | **<0.001** | **0.25** |

*Values are presented as mean ± standard deviation or median (IQR) for continuous variables and as number and % for categorical variables. ** p value according to ANOVA/Kruskal-Wallis test for continuous variables and Chi-square for categorical variables. Bonferroni correction was used.

^a^ **Diaphragmatic no obstruction/ gangrene vs. Inguinal Hernia no obstruction/ gangrene**

^b^ **Diaphragmatic no obstruction/ gangrene vs. Inguinal Hernia with obstruction/ gangrene**

^c^ **Diaphragmatic no obstruction/ gangrene vs. Ventral Hernia no obstruction/ gangrene**

^d^ **Diaphragmatic no obstruction/ gangrene vs. Ventral Hernia with obstruction/ gangrene**

^e^ **Inguinal Hernia no obstruction/ gangrene vs. Ventral Hernia no obstruction/ gangrene**

^f^ **Inguinal Hernia no obstruction/ gangrene vs. Ventral Hernia with obstruction/ gangrene**

^g^ **Inguinal Hernia with obstruction/ gangrene vs. Ventral Hernia non obstruction/ gangrene**

^h^ **Inguinal Hernia with obstruction/ gangrene vs. Ventral Hernia with obstruction/ gangrene**

^i^ **Ventral Hernia with obstruction/ gangrene vs. Ventral Hernia no obstruction/ gangrene**

^j^ **Inguinal Hernia non obstruction/ gangrene vs. Inguinal Hernia with obstruction/ gangrene**

**Supplemental Table 2.** Hernia prevalence and odd ratios across BMI categories

| BMI categories | Entire (n) | % of entire population | non-Hernia (n) | Hernia (n) | % Hernia | OR (CI) |
| --- | --- | --- | --- | --- | --- | --- |
| Underweight | 54,286 | 3.984 | 53,692 | 594 | 1.094 | 0.55  (0.51-0.60) |
| Normal weight | 527,997 | 38.754 | 517,582 | 10,415 | 1.973 | REF |
| Overweight | 454,793 | 33.381 | 443,238 | 11,555 | 2.541 | 1.30  (1.26-1.33) |
| Obesity | 274,687 | 20.161 | 267,316 | 7,371 | 2.683 | 1.37  (1.33-1.41) |
| Morbid obesity | 50,677 | 3.720 | 49,555 | 1,122 | 2.214 | 1.13  (1.06-1.20) |
| Total | 1,362,440 | 100 | 1,331,383 | 31,057 | 2.280 |  |

**Supplemental Table 3:** Hernia prevalence and odd ratios across BMI categories

| BMI categories | Underweight | Normal weight | Overweight | Obesity | Morbid obesity | Super morbid obesity | Total |
| --- | --- | --- | --- | --- | --- | --- | --- |
| Entire(n) | 54,286 | 527,997 | 454,793 | 274,687 | 43,028 | 7,649 | 1,362,440 |
| %of entire population | 3.984 | 38.754 | 33.381 | 20.161 | 3.158 | 0.561 | 100 |
| All hernia types | | | | | | | |
| non-Hernia (n) | 53,692 | 517,582 | 443,238 | 267,316 | 42,057 | 7,498 | 1,331,383 |
| Hernia (n) | 594 | 10,415 | 11,555 | 7,371 | 971 | 151 | 31,057 |
| Hernia prevalence (per 10,000) | 109.4 | 197.2 | 254.1 | 268.3 | 225.7 | 197.1 | 228.0 |
| OR (CI) | 0.55  (0.51-0.60) | REF | 1.30  (1.26-1.33) | 1.37  (1.33-1.41) | 1.15  (1.07-1.23) | 1.00  (0.85-1.18) |  |
| Diaphragmatic without obstruction/  gangrene | | | | | | | |
| Diaphragmatic without obstruction/  Gangrene (n) | 356 | 4,794 | 5,088 | 3452 | 461 | 62 | 14,213 |
| Prevalence Diaphragmatic without obstruction/  gangrene (per 10,000) | 65.6 | 90.8 | 111.9 | 125.7 | 107.1 | 81.1 | 104.3 |
| OR (CI) | 0.72  (0.65-0.80) | REF | 1.23  (1.19-1.28) | 1.39  (1.33-1.45) | 1.18  (1.07-1.30) | 0.89  (0.69-1.15) |  |
| Ventral hernia without obstruction/  gangrene | | | | | | | |
| Ventral hernia without obstruction/  gangrene (n) | 111 | 2218 | 3140 | 2775 | 410 | 81 | 8735 |
| Prevalence ventral hernia without obstruction/  gangrene (per 10,000) | 20.5 | 42.0 | 69.0 | 101.0 | 95.3 | 105.9 | 64.1 |
| OR (CI) | 0.49  (0.40-0.59) | REF | 1.65  (1.56-1.74) | 2.42  (2.29-2.56) | 2.28  (2.05-2.54) | 2.54  (2.03-3.17) |  |
| Inguinal without obstruction/  gangrene | | | | | | | |
| Inguinal without obstruction/  gangrene (n) | 109 | 3221 | 3129 | 1016 | 77 | 4 | 7556 |
| Prevalence inguinal without obstruction/  gangrene (per 10,000) | 20.1 | 61.0 | 68.8 | 37.0 | 17.9 | 5.2 | 55.5 |
| OR (CI) | 0.33  (0.27-0.40) | REF | 1.13  (1.07-1.19) | 0.60  (0.56-0.65) | 0.29  (0.23-0.37) | 0.09  (0.03-0.23) |  |

**Supplemental Table 4.** Hernia prevalence and odd ratios across BMI categories among women

| BMI categories | Underweight | Normal weight | Overweight | Obesity | Morbid obesity | Super morbid obesity | Total |
| --- | --- | --- | --- | --- | --- | --- | --- |
| Entire(n) | 41250 | 335833 | 220560 | 145959 | 27864 | 5181 | 776647 |
| %of entire population | 5.31 | 43.24 | 28.40 | 18.79 | 3.59 | 0.67 | 100.00 |
| All hernia types | | | | | | | |
| non-Hernia (n) | 40828 | 330986 | 216766 | 142666 | 27242 | 5075 | 763563 |
| Hernia (n) | 422 | 4847 | 3794 | 3293 | 622 | 106 | 13,084 |
| Hernia prevalence (per 10,000) | 102.3 | 144.3 | 172.0 | 225.6 | 223.2 | 204.6 | 168.5 |
| OR (CI) | 0.71 (0.64-0.78) | REF | 1.2 (1.15-1.25) | 1.58 (1.51-1.65) | 1.56(1.43-1.70) | 1.43(1.17-1.73) |  |
| Diaphragmatic without obstruction/  gangrene | | | | | | | |
| Diaphragmatic without obstruction/  Gangrene (n) | 277 | 2991 | 2593 | 2233 | 369 | 53 | 8516 |
| Prevalence Diaphragmatic without obstruction/  gangrene (per 10,000) | 67.2 | 89.1 | 117.6 | 153.0 | 132.4 | 102.3 | 109.7 |
| OR (CI) | 0.75(0.66-0.85) | REF | 1.32(1.26-1.40) | 1.73(1.64-1.83) | 1.49(1.34-1.67) | 1.15(0.88-1.51) |  |
| Ventral hernia without obstruction/  gangrene | | | | | | | |
| Ventral hernia without obstruction/  gangrene (n) | 88 | 1289 | 967 | 914 | 228 | 50 | 3536 |
| Prevalence ventral hernia without obstruction/  gangrene (per 10,000) | 21.3 | 38.4 | 43.8 | 62.6 | 81.8 | 96.5 | 45.5 |
| OR (CI) | 0.55 (0.45-0.69) | REF | 1.14(1.05-1.24) | 1.64(1.50-1.78) | 2.14(1.86-2.47) | 2.53(1.90-3.36) |  |
| Inguinal without obstruction/  gangrene | | | | | | | |
| Inguinal without obstruction/  gangrene (n) | 47 | 480 | 191 | 104 | 10 | 1 | 833 |
| Prevalence inguinal without obstruction/  gangrene (per 10,000) | 11.4 | 14.3 | 8.7 | 7.1 | 3.6 | 1.9 | 10.7 |
| OR (CI) | 0.80 (0.59-1.08) | REF | 0.61(0.51-0.72) | 0.50(0.40-0.62) | 0.25(0.13-0.47) | 0.13(0.02-0.95) |  |

**Supplemental Table 5:** Hernia prevalence and odd ratios across BMI categories among men

| BMI categories | Underweight | Normal weight | Overweight | Obesity | Morbid obesity | Super morbid obesity | Total |
| --- | --- | --- | --- | --- | --- | --- | --- |
| Entire(n) | 13685 | 203626 | 242133 | 131441 | 15645 | 2572 | 609102 |
| %of entire population | 2.25 | 33.43 | 39.75 | 21.58 | 2.57 | 0.42 | 100.00 |
| All hernia types | | | | | | | |
| non-Hernia (n) | 13513 | 198059 | 234372 | 127364 | 15297 | 2527 | 591132 |
| Hernia (n) | 172 | 5567 | 7761 | 4077 | 348 | 45 | 17970 |
| Hernia prevalence (per 10,000) | 102.3 | 144.3 | 172.0 | 225.6 | 223.2 | 204.6 | 295.0 |
| OR (CI) | 0.45 (0.39-0.53) | REF | 1.18 (1.14-1.22) | 1.14 (1.09-1.19) | 0.81 (0.73-0.90) | 0.63(0.47-0.85) |  |
| Diaphragmatic without obstruction/  gangrene | | | | | | | |
| Diaphragmatic without obstruction/  Gangrene (n) | 79 | 1802 | 2495 | 1218 | 91 | 9 | 5694 |
| Prevalence Diaphragmatic without obstruction/  gangrene (per 10,000) | 57.7 | 88.5 | 103.0 | 92.7 | 58.2 | 35.0 | 93.5 |
| OR (CI) | 0.65(0.52-0.82) | REF | 1.17 (1.10-1.24) | 1.05(0.97-1.13) | 0.66(0.53-0.81) | 0.39(0.20-0.76) |  |
| Ventral hernia without obstruction/  gangrene | | | | | | | |
| Ventral hernia without obstruction/  gangrene (n) | 23 | 929 | 2173 | 1861 | 182 | 31 | 5199 |
| Prevalence ventral hernia without obstruction/  gangrene (per 10,000) | 16.8 | 45.6 | 89.7 | 141.6 | 116.3 | 120.5 | 85.4 |
| OR (CI) | 0.37 (0.24-0.56) | ref | 1.98(1.83-2.13) | 3.13(2.90-3.39) | 2.57(2.19-3.01) | 2.66 (1.86-3.82) |  |
| Inguinal without obstruction/  gangrene | | | | | | | |
| Inguinal without obstruction/  gangrene (n) | 62 | 2741 | 2938 | 912 | 67 | 3 | 6723 |
| Prevalence inguinal without obstruction/  gangrene (per 10,000) | 45.3 | 134.6 | 121.3 | 69.4 | 42.8 | 11.7 | 110.4 |
| OR (CI) | 0.33 (0.26-0.43) | REF | 0.90(0.85-0.95) | 0.51(0.47-0.55) | 0.32(0.25-0.40) | 0.09(0.03-0.26) |  |
